# Post-treatment surveillance in colorectal cancer

**DOI:** 10.2478/v10019-010-0018-8

**Published:** 2010-09-09

**Authors:** Vaneja Velenik

**Affiliations:** Institute of Oncology Ljubljana, Ljubljana, Slovenia

**Keywords:** surveillance, colorectal cancer

## Abstract

**Background:**

Though the post treatment surveillance of patients with colorectal cancer (CRC) treated with curative intent is common practice, its value is controversial. In the absence of conclusive clinical data, various modalities for the routine follow-up of patients with CRC have been proposed. In practice, the guidelines across countries and regions differ and are influenced by different health care policies, resource availability and doubts about effectiveness of follow-up.

**Conclusions:**

The results of metaanalyses of available clinical trials demonstrated a survival benefit of intensified monitoring, but the questions regarding the optimal frequency of visits and the examinations to be performed remain unanswered. Furthermore, intensive monitoring of CRC survivors may be difficult to be administrated, causes discomfort and morbidity to the patient and can have serious cost-implications to the healthcare system. However, as it seems from available data, a comprehensive surveillance program does not affect the quality of patients’ life. Ongoing large prospective multi-institutional randomised trials might elucidate some of the crucial questions and existing dilemmas to establish adequate surveillance strategy for CRC patients.

## Introduction

Colorectal cancer (CRC) is a significant public health problem. In Slovenia, CRC is the second most frequently diagnosed cancer in both men and women and the second leading cause of cancer death, with estimated 1,284 new cases and 682 related deaths in 2006.^1^ Five year relative survival in 2005 was 57.7% for colon cancer and 45.4% for rectal cancer, increasing by 16.2% and 11.4%, respectively, from 1991.^2^ Over the last two decades, CRC research has lead to better understanding of disease behaviour, resulting in more efficient treatments and higher prevalence of cancer survivors. In spite of radical treatment, approximately 30–50% of patients will develop recurrent disease of whom only 5–30% would be considered eligible for further surgery; of those only 3–5% will be actually cured.^3^,^4^ In addition, the reported rates of second primary tumours in CRC patients are ranging from 5% to 10%.^5^–^10^ Furthermore, long-term analyses of Scandinavian trials have shown an increased risk of second cancers in the patients treated with preoperative radiotherapy for rectal cancer in organs within or adjacent to the irradiated volume.^11^

The main aim of post-treatment surveillance after potentially curative treatment of CRC is to improve survival through early detection of polyps and new primaries or recurrent tumours when efficient treatment is possible.^12^–^15^ Secondary goals are to assess the efficacy of initial treatment, management of long-term post-treatment complications, to offer comprehensive psychologic support and support in disease prevention.^16^

## Studies of CRC follow-up strategies

To define the value of varying levels of follow-up intensity in surveillance programs among CRC survivors, six randomized controlled trials were conducted (Table 1).^17^–^22^ Two of them have showed a survival benefit from more intensive follow-up.^21^,^22^ There was a great variability between the studies in defining the follow-up. For example, the kind of follow-up that was considered as “intensive” in the study by Makela *et al.*^17^, was assessed by Shoemaker *et al.*^20^ as “less intensive”. In some of the studies, the sample size was not sufficient to detect survival differences with different surveillance strategies, and some of the studies included patients with stage I disease.

Therefore, some meta-analysis were performed as a systematic approach to identification and abstraction of critical information from different randomised, controlled trials.^23^ Two meta-analyses of five randomised trials identified a survival advantage for the patients followed more intensely as significantly higher incidence of asymptomatic local or systemic recurrence was recognized among the patients monitored closely and, consequentially, reoperation for cure was more frequent in this group.^24^,^25^ These results were confirmed by another, recently published meta-analysis including six randomised trials on this topic with a significant improvement in survival favouring more intense follow-up (Relative Risk Ratio 0.80; 95% CI, 0.70 to 0.91; p = 0.0008). A significant improvement in survival was observed only those trials which included CEA testing and/or liver imaging.^26^ Another two meta-analyses (on randomised and nonrandomised trials) concluded that intensive follow-up programmes can improve survival^27^,^28^, and should be »individualised« according to a person’s characteristics.^27^

In an attempt to rationalize CRC follow-up, three prospective multi-institutional randomised trials comparing more intensive with less intensive monitoring are being carried out at the moment: the FACS trial in United Kingdom, the FFCD trial in France and the GILDA trial in Italy.^29^ The GILDA follow-up schemes are presented in Table 2. The results of these trials are pending.

## Potential limitations of follow-up

Few considerations have to be taken into account when promoting surveillance and there are some limitations to this approach.

First, there is a small risk of adverse events associated with colonoscopy itself or with polipectomy during the follow-up. Only one of the prospective randomised follow-up studies reported these data: two perforations and two gastrointestinal haemorrhages from a total of 731 colonoscopies.^20^

Secondly, frequent visits to physician might be inconvenient to the patients and even harmful due to unnecessary exposition to radiation.^30^ Fear of recurrence or unnecessary stress resulting from false positives results may also have a negative impact on the quality of their lives. False positive results are on average 16 times (0.2–200) more common than true positive results.^31^ On the other hand, reassuring effect of normal test results and psychological support from physician might be beneficial. The data about the effect of follow-up on patients’ health-related quality of life (HRQL) are limited and conflicting. While Stiggelbout *et al*. and Wattchow *et al*. indicated that HRQL was not improved through follow-up visits, Kjeldsen *et al*. demonstrated a small but significant increase in HRQL with a more intensive follow up.^32^–^34^ However, Stiggelbout *et al*. emphasized that most patients would prefer regular contacts even if they showed no benefit in terms of earlier detection of recurrence.^32^

The third factor to be taken into consideration when promoting surveillance is high cost of such program. A wide variety of follow-up schemes are associated with large differences in costs. Few studies focused on this issue. Virgo *et al*. reported a 28-fold difference in costs between minimal and most extensive 5-year follow-up in USA, ranging from US$ 910 to US$ 26.717.^35^ Audisio *et al*. calculated the 5-year follow-up costs in Italy as follows: US$ 3.800 per patient; US$ 13.580 for each recurrence; US$ 59.841 for every recurrence treated for cure and US$ 13.6779 for each cured patient; the difference in costs between minimal and aggressive 5-year follow-up protocol was US$ 4.800 per patient. Authors recommended that the programmes should be tailored to the stage and site of primary cancer in order to reduce costs^36^ and that controlled economic studies are required.^37^ The cost-effectiveness analysis of five randomized trials showed that the cost for the intensive follow-up resulted in a net extra cost of US$ 4.214–4.299 per patient compared with the less intensive follow-up arm. Each life year saved through the intensive follow-up was calculated to cost between US$ 5.230–5.783.^24^

When resectability of recurrences was considered, a cost minimization analysis performed by Rodrigues *et al*. demonstrated that the cost per resectable tumour recurrence was lower in the intensively followed group.^38^ This is a logical conclusion despite the fact that the overall cost of intensive follow up was higher in the intensive strategy group than in less intensive one.

Other authors pointed to the high cost of follow-up suggesting that it should be transferred to the primary care setting. The arguments were that the specialist care is more intense and that specialists tend to propose more expensive follow-up strategies.^39^

The question remains, who should carry out the follow-up visits. With increasing numbers of CRC survivors, primary care physicians (PCPs) are more and more engaged in CRC follow-up programs.^40^–^42^ The data from the literature regarding the utility of general versus specialist care in CRC survivors are sparse. In a study by Nissen *et al*., PCPs reported dissatisfaction with this transfer of care for survivors; they also felt uncertain about the appropriate frequency and duration of surveillance testing for cancer recurrence.^43^ Moreover, in a recently published study by Snyder *et al*., the authors reported a decreased intensity of cancer-related screening program as oncologists were becoming less involved in survivor care. The survivors followed up by both a PCP and an oncologist were most likely to receive both noncancer-related recommended care and cancer surveillance.^42^ The authors concluded that a shared model of survivorship care should be developed with a clear and detailed description of roles of both sides, PCP’s and oncologist’s, to gain maximal coordination and efficacy.^44^ On the other hand, some data suggest that the survivors followed up by PCP only did not perceive lower quality of care^40^, which was also confirmed by others mentioning that no difference was recorded in the rate of recurrence and death as well as time to detection of recurrence in comparison to the patients followed by a surgeon or PCP.^45^

Finally, with respect to cost and time consumption of follow-up, it seems reasonable that the surveillance of patients for whom additional therapeutic options when recurrence occurs are available^4^,^46^, should be more intense. Furthermore, particular attention was paid to determine the subgroups of CRC patients which might benefit the most from follow-up with regard to tumour site or stage. The results of a prospective randomized trial on 259 CRC survivors conducted by Rodrigues-Moranta *et al*. indicated that the patients with stage II tumours or lesions in the rectum had higher overall survival when followed more intensively than those on less intensive follow-up program. No difference was found between the patients with stage III lesions or lesions located in colon.^38^

## Current recommendations and adherence to them by physicians

Several guidelines have been published on the surveillance of CRC survivors. Follow-up program is recommended by all leading professional societies, *e.g*. the American Society of Clinical Oncology (ASCO)^47^, National Comprehensive Cancer Network (NCCN)^48^,^49^ and European Society Medical Oncology (ESMO).^50^,^51^ Surveillance protocols include regular outpatient’s visits followed by physical examination, CEA monitoring, radiological and endoscopic examinations. It must be stressed that none of diagnostic procedures by itself is sensitive or specific enough to detect the recurrence at early, treatable stage; so, the guidelines recommend different packages of tests.

Although there are differences in frequency, intensity and combinations of investigations as proposed by various programs, some parts of recommendations are similar (Table 3). Monitoring is more intense during the first two to three years after radical treatment, as most of the recurrences occur within this period of time. There is a debate when to stop performing individual tests or monitoring the patients as they are an increased lifetime risk of developing recurrent disease or new primary CRC. The guidelines recommend continued, albeit less frequent visits during 3–5 years after therapy. Although local recurrences after adjuvant therapy are less common, irradiated rectal cancer patients may experience late relaps.^52^,^53^ Many experts believe that follow-up beyond five years is necessary for such patients.^54^

While recommendations concerning colonoscopy in high risk patients are consistent, there is a great variability in the standards of other tests. In ESMO guidelines the routine clinical, laboratory and radiological examinations are not indicated in rectal cancer patients at all. Furthermore, pelvic CT scanning for rectal cancer is recommended only in ASCO guidelines (Table 3).

Due to aggressive therapy, CRC survivors can exhibit late *sequel* of treatment^54^–^59^, most common being impaired bowel, voiding, sexual malfunctioning and quality of life impairment, bone fractures after pelvic radiation, oxaliplatin-induced neuropathy and *psychosocial distress.* Among the three guidelines mentioned only the NCCN ones describe potential late effects of treatment; unfortunately, they include only little information on how to manage the symptoms.

The consequence of lacking uniform guidelines is that the heterogeneity among physicians regarding the use of follow-up tests is serious. For example, a postal survey of all the active members of the American Society of Colon and Rectal Surgeons (ASCRS) undertaken in 1994 found that only 50% of surgeons who returned the questionnaire adhere to official recommendations.^60^ Twelve years later, Giordano *et al*. reported that only 30% of surgeons followed any guidelines. Of these, only 20% stuck to the national guidelines and 80% followed local recommendations.^61^

Disparities in follow-up care are also observed when patient’s race and age are taken into account. The report from Rolnick *et al*. highlighted that one of the reasons for poorer survival of black CRC patients in comparison with white patients might be that they have less follow-up surveillances.^62^ A study conducted by Cooper *et al*. in 9.426 patients aged over 65 revealed that less than half of older CRC patients in the US during post-therapy period receive recommended screening for recurrence, indicating that the physician’s preferences may influence the choice of testing.^63^

## Conclusions

Follow-up of CRC survivors is a common practice. Intensive surveillance enhances the probability of diagnosing precancerous lesions, recurrences or new primaries at early stage when the existing treatment options could be used with curative intent. Consequentially, comprehensive surveillance program improves the survival and at the same time – as it seems from available data, does not affect the quality of patients’ life. On the other hand, the increased costs and time consumption of intensive surveillance limit its utility. Due to limited, and to some extent conflicting data, there are no uniform guidelines for the CRC survivors regarding the frequency of visits and tests to be performed at each visit. Ongoing large prospective multi-institutional randomised trials might elucidate some of the crucial questions and existing dilemmas to establish an adequate surveillance strategy for CRC patients.

## Figures and Tables

**TABLE 1:** Studies comparing intensive with less intensive follow-up

**Studies**	**Year**	**No**	**CEA testing**	**Liver imaging**	**5-y OS IFU (less IFU)**	**P value**
**Makela^17^**	1995	106	Yes	Yes	59 (54)	0.26
**Ohlsson^18^**	1995	107	Yes	No	75 (67)	0.50
**Kjeldsen^19^**	1997	597	No	No	68 (70)	0.48
**Schoemaler^20^**	1998	325	No	Yes	76 (70)	0.20
**Pietra^21^**	1998	207	Yes	Yes	73 (58)	0.02
**Secco^22^**	2002	358	Yes	Yes	62 (43)	<0.05

Abbreviations: No = number of patients; OS = overall survival; IFU = intensive follow-up

**TABLE 2:** GILDA trial for rectal cancer follow-up

	**Months from randomisation**										
	**4**	**8**	**12**	**16**	**20**	**24**	**30**	**36**	**42**	**48**	**60**

**Less intensive**											
Office visit	+	+	+	+	+	+	+	+	+	+	+
CEA	+	+	+	+	+	+	+	+	+	+	+
Proctoscopy	+										
Colonoscopy			+							+	
Chest X-ray			+								
Liver ultrasound		+		+							
**More intensive**											
Office visit	+	+	+	+	+	+	+	+	+	+	+
Blood tests	+	+	+	+	+	+	+	+	+	+	+
Proctoscopy	+	+									
Colonoscopy			+			+		+		+	+
Chest X-ray			+			+		+		+	+
Liver ultrasound	+	+	+	+		+		+		+	+
Abdominal-pelvic CT	+		+			+				+	

Abbreviations: blood tests include complete blood count, liver tests, tumour markers CEA and Ca 19-9.

**TABLE 3:** Follow-up guidelines of main professional societies

**Modality**	**ASCO^47^**	**NCCN^48^,^49^**	**ESMO^50^,^51^**
History, physical exam	Every 3–6 m for 3 y, then every 6 m up to 5 y	Every 3–6 m for 2 y, then every 6 m up to 5 y	every 3–6 m for 3 y, then every 6–12 m for 2 y (colon) every 6 m for 2 y (rectal cancer)
Colonoscopy	at 3y, every 5y thereafter	At 1y, then at 3y, every 5y thereafter	After 1y, then every 3y (colon) every 5y (rectal cancer)
Flexible proctoscopy (rectal cancer)	every 6m for 5y (for not irradiated patients)	every 6m for 5y (for patients with LAR)	every 6 m for 2 years
Blood tests	not recommended	not recommended	not recommended
CEA	every 3–6m for 3y (stage II and III)	every 3–6m for 2y, then every 6m up to 5y (staged as T2 or greater)	if initially elevated: every 3–6m for 3y, then every 6–12m for 2y (colon) not recommended (rectal cancer)
Chest x-rays	not recommended	not covered	not recommended
US abdomen	not covered	not covered	not recommended
CT thorax and CT abdomen	annually for 3y for pts with high risk of recurrence	annually for 3–5y for stage II and III	Every 6m for 3y for pts with high risk of recurrence (colon) Not recommended (rectal cancer)
Pelvic CT (rectal cancer)	negative prognostic features, especially for not irradiated pts (no frequency)	Not covered	not recommended

Abbreviations: m=months; y=years; ASCO=American Society Clinical Oncology; NCCN=National Comprehensive Cancer Network; ESMO=European Society Medical Oncology
